# Effect of caloric restriction on depression

**DOI:** 10.1111/jcmm.13418

**Published:** 2018-02-21

**Authors:** Stephen Malunga Manchishi, Ran Ji Cui, Xiao Han Zou, Zi Qian Cheng, Bing jin Li

**Affiliations:** ^1^ Jilin Provincial Key Laboratory on Molecular and Chemical Genetics The Second Hospital of Jilin University Changchun Jilin China; ^2^ Department of Physiology University of Cambridge Cambridge UK

**Keywords:** antidepressant, fasting, calorie restriction, BDNF

## Abstract

Recently, most of evidence shows that caloric restriction could induce antidepressant‐like effects in animal model of depression. Based on studies of the brain–gut axis, some signal pathways were common between the control of caloric restriction and depression. However, the specific mechanism of the antidepressant‐like effects induced by caloric restriction remains unclear. Therefore, in this article, we summarized clinical and experimental studies of caloric restriction on depression. This review may provide a new therapeutic strategy for depression.

**Table:** 

• **Introduction**
**• Antidepressant effects of caloric/dietary restriction**
**• Antidepressant effects of fasting**
**• Concluding remarks**
**• Acknowledgements**
**• Conflict of interest**

## Introduction

Depression is the most commonly diagnosed neuropsychiatric disease with symptoms including depressed mood, loss of interest or pleasure in previously pleasurable activities, disturbed sleep and/or appetite, self‐injury and/or suicidal thoughts 1, 2. Mortality is relatively high among patients with bipolar disorder and depression due to increased risk for suicide 3. A Global Burden of Disease study showed depression is the most disabling disorder worldwide 4. Despite the number of available of antidepressant drugs, their limited efficacy in addition to their serious adverse effects remains a major concern and necessitates the continued search and validation of new therapeutic interventions 5, 6.

The common pathological pathways of depression and obesity suggest a possible link between genes involved in the regulation of food intake and depression 7, 8. Both depression and obesity share irregularities in a myriad of neurotransmitters, neuropeptides, cytokines, receptor molecules, enzyme systems and transcription factors, all resulting in the dysregulation of hypothalamic–pituitary–adrenal (HPA) axis 9, 10, 11, 12, 13. The HPA axis has been found abnormal, believed to be related in part to an impaired function of the glucocorticoid receptors (GR) and by promoting oligodendrogenesis, in psychiatric disorders including major depression 14, 15. This is demonstrated by significant increase in cortisol in depressed patients. At their normal levels, glucocorticoids regulate many physiological aspects including neuronal survival and neurogenesis 16. However, a sustained abnormal increase can cause alterations in dendrite and spine morphology in specific brain regions, and eventually suppress brain derived neurotrophic factor (BDNF) and adult neurogenesis 17, 18. Suppressed neurogenesis and neuronal atrophy have been implicated in depressive disorders 14.

A search for antidepressant activity among edible products using animal models of depression has been on the upswing. Discovery of anti‐depressive foods is attractive as it could have considerable impacts on the fight against depression worldwide 19. Diet is one of the non‐invasive approaches that can be used to enhance neural signalling by influencing synaptic transmission and brain plasticity 20. Recent studies point to dietary factors as important effectors in the brain, suggesting a direct relationship with psychiatric disorders including, major depressive disorder and anxiety 21. Several dietary compounds included in edible products as well as reduced calorie intake and fasting, without falling into malnutrition, have a broad and positive action on a range of molecular systems supporting neuronal function and plasticity, exerting antidepressant‐like effects 6, 22. On the other hand, poor dietary practices, typical of our modern society, are considered risk factors for various neurodegenerative diseases 23.

By comparison with the large amount of data suggesting the beneficial effects of reduced energy intake on longevity, cognitive function, memory, cardiovascular disease, cancers, chronic pain and other neurologic disorders, relatively little is experimentally known of the direct influence of reduced energy intake on depression. Most of what is known is a result of findings of studies focusing on other disorders and extrapolation based on mechanisms of action and the known pathophysiology of depression. Beneficial effects of energy restriction include orexin signalling activation, CREB/BDNF signalling activation, endorphin release and production of ketone 1. Other mechanisms that may underlie the efficacy of energy restriction include stimulation of autophagy, a lysosomal degradative process which recycles cellular waste and eliminates potentially toxic damaged organelles and protein aggregates. Autophagy dysregulation results in a number of neurodegenerative disorders while up‐regulation of this pathway may be neuroprotective 24, 25.

In this article, we discuss current knowledge of energy restriction in alleviating depression by reviewing both animal studies and clinical findings. We also explore dietary supplements from natural sources for the prevention and attenuation of symptoms of depression.

## Antidepressant effects of caloric/dietary restriction

Caloric restriction has attracted increasing attention due to its evident effects on neuroendocrine system and mood condition 1. Defined as the reduction in caloric intake without malnutrition, caloric restriction has beneficial effects described at both organismal and cellular levels 26. Both animal and human studies have shown that caloric restriction increases longevity, memory, quality of life and reduces risk factors for neurodegenerative and psychiatric diseases including depression 27, 28, 29, 30. Physicians allow patients to drink water *ad libitum* while consuming very low‐calorie food for a week or more 20 and has showed no negative, but some positive effects on health‐related quality of life including mood, sleep and sexual function in both men and women 31. Animal studies, especially in rodents have also proved the beneficial effects of caloric and dietary restriction in longevity and in models of psychiatric and neurodegenerative disorders 32, 33, 34, 35.

Decreased neurogenesis has been implicated in the pathogenesis of anxiety and depression. Hippocampal neurogenesis presents a potential new strategy for treating depression. Interestingly, reducing the number of calories consumed promotes the survival of newly generated cells in the hippocampus 36, 37. Furthermore, chronic mild food restriction activates AMPK following decreased hypothalamic malonyl‐CoA, an inhibitor of fatty acid oxidation 38.

Reduced cerebral blood flow is also linked to anxiety and depression. Caloric restriction has been shown to enhance cerebral blood flow and blood–brain barrier function in young mice at 5–6 months of age and is protective for cerebral blood flow in old adult rodents. The neurovascular enhancements were associated with reduced mammalian target of rapamycin (mTOR) expression, similar to the effects of the antidepressant ketamine 39.

Acute, short‐term and long‐term caloric restrictions have all been shown to activate the HPA axis, increasing the levels of glucocorticoids and ameliorate depressive symptoms in a hormetic manner 40. The mechanism by which this increase in glucocorticoids induces neuronal survival and enhances BDNF is not entirely known. In mice, acute caloric restriction rapidly approaches the effects of long‐term caloric restriction, suggesting that the beneficial effects of caloric restriction may require only a short‐term reduction in caloric intake 41, 42. Moreover, the beneficial effects induced by a short period of dietary restriction in adult mice were retained even when *ad libitum* feeding was reintroduced, though under continuous dietary restriction, lifespan extension was more prominent in females than in males 43, 44. However, in another study, 90% of the gene expression effects of long‐term caloric restriction were reversed within 8 weeks of return to *ad libitum* feeding 45.

## Antidepressant effects of fasting

Periodic fasting voluntarily for religious or cultural purposes or involuntarily due to lack is a common practice around the world. Medical or therapeutic fasting is also practiced and has been shown to be safe 13. Fasting acutely increases levels of the orexigenic gastrointestinal hormone, acyl‐ghrelin. Ghrelin is a growth hormone secretagogue receptor (Ghsr) ligand within the hippocampus, increasing dentate gyrus levels of the neurogenic transcription factor Egr‐1, enhancing adult hippocampal neurogenesis 26.

We have previously shown that acute fasting produces antidepressant‐like effects in mice, accompanied by an increase in catecholamines and glucocorticoids 42. To protect itself from the potentially deleterious effects of these hormones, the brain's cellular mechanisms of stress resistance that could promote neurogenesis, synthesis of neurotrophic factors, receptors for neurotransmitters and chaperone proteins are activated 13. Short‐term fasting leads to a dramatic up‐regulation in neuronal autophagy (sometimes referred to as cellular cleansing) characterized by diminished neuronal mTOR activity *in vivo* 25. Studies in rodent models showed that fasting enhanced the availability of brain tryptophan and serotonin 46. 5–10 days of fasting in humans or 1–2 days in rats reportedly induces releases of endogenous endorphins, which could explain mood improvements with no correlations in weight loss 13. Other studies have showed that intermittent fasting causes an increase in BDNF, which is one of the known neurotrophic factors involved in many antidepressants (Table 1) 47.

**Table 1:** Effects of energy restriction on the brain's potential indicators of antidepressant effect

Energy restriction type	Effect/mechanism of action	References
Calorie/Dietary restriction	Activation of orexin neurons	48
	Increase in BDNF	35, 49, 50, 51
	Diminished production of mitochondrial reactive oxygen species (ROS)	52, 53
	Increase in heat‐shock protein	54, 55
	Production of ketone bodies	56
	Increased CREB phosphorylation	57
Acute fasting	Increased CREB phosphorylation, reduced immobility in FST, increased corticosterone.	42
	Enhanced autophagy	25
Intermittent fasting	Increase in BDNF	32, 58
Ramadan intermittent fasting	Reduction of proinflammatory cytokines IL‐1β, IL‐6 and TNFα and body fat percentage	59

## Mechanism of action

The most probable mode of action of energy restriction on neurological disorders is *via* BDNF signalling. BDNF, a member of the neurotrophin family, is related to brain health, food intake and glucose metabolism 48, and it can cross the blood–brain barrier in both directions 49. The action of BDNF is crucial for supporting cognitive abilities, and dysfunction or reductions in BDNF levels have been proposed to be part of the pathobiology of various neurological disorders 23. Conversely, increased BDNF level has been associated with reduced symptoms of depression. BDNF is a potent ligand for the tyrosine receptor kinase B (TrkB) 50, activating protein kinase B (Akt), phospholipase C gamma (PLCγ), and cAMP response element‐binding protein (CREB) signalling pathways 51 which mediates several effects such as growth and differentiation of neurons and synaptic plasticity and synaptic transmission, neurogenesis and neuronal survival in the adult brain 20.

BDNF/CREB pathway is a pharmacological target of many neurological disorder therapies. Guzzardi *et al*. suggest that a safer approach of targeting this pathway may be to identify and optimize mediators that indirectly promote BDNF production 48. Dietary restriction has been shown to increase levels of BDNF 36, 52, leading to hippocampal neurogenesis 53 and subsequent behavioural improvements. Rather than affecting proliferation of neuronal pluripotent cells per se, it enhances neurogenesis by increasing the survival of newly generated cells 36. On the other hand, glycemia and high‐fat diet have been shown to be negative predictors of BDNF levels 48. In high‐fat diet fed mice, serum BDNF levels were beyond the detectable range, while BDNF mRNA expression was inhibited in the ventral medial hypothalamus 54. The behavioural improvements caused by BDNF were abolished in mice lacking CREB 55, suggesting that CREB is a critical, indispensable link in the pathway. As a matter of fact, some experiments of energy restriction have reported an increase in CREB phosphorylation, but not serum BDNF levels 42. Therefore, BDNF signalling plays important roles in regulating adult hippocampal neurogenesis under basal conditions and in response to dietary restriction 53. Moreover, TrkB was increased in the hippocampus during dietary restriction, which would be expected to result in increased BDNF‐TrkB signalling 36.

The mechanism by which dietary restriction increases BDNF is not entirely known, but it could be a mild metabolic stress response in neurons 36 as in ketogenic therapy. Ketogenic therapy shifts the body into a state of ketone body production 56. Fasting is a strong physiological stimulus equivalent to a biological stress that activates the hypothalamic–pituitary–adrenal axis 13. Fasting and low‐carbohydrate/high‐fat diet were found to increase levels of the ketone bodies β‐hydroxybutyrate and acetoacetate in normal, healthy individuals (reviewed in 56) while the deacetylase inhibition properties of ketones increase the expression of BDNF 56, 57, 58.

Two antagonistic peptide hormones, leptin and ghrelin, have been reported to affect mental status 5. Ghrelin is a hormone produced primarily by distinct ghrelin cells located in gastrointestinal tract, while leptin is a hormone derived from adipocytes. Both hormones are transported across the blood–brain barrier to exert their central effects. Fasting acutely increases levels of acyl‐ghrelin, increasing dentate gyrus levels of the neurogenic transcription factor Egr‐1, enhancing adult hippocampal neurogenesis 26, 59, 60, 61. Fasting for over 8 days decreased leptin levels.

Other mechanisms that may underlie the efficacy of energy restriction include stimulation of autophagy, a lysosomal degradative process which recycles cellular waste and eliminates potentially toxic damaged organelles and protein aggregates 24, 25, 62, reduction of proinflammatory cytokines 63, diminished production of mitochondrial reactive oxygen species (ROS) 64, 65 and increase in heat‐shock proteins, in what is seen as a preconditioning mechanism to increase resistance of the neurons to subsequent insults 66, 67. Levels of heat‐shock protein‐70 were significantly increased in cortical synaptosomes from dietary restricted rats compared with rats fed *ad libitum* 66.

Most antidepressants also affect body weight, with most of them inducing weight gain and only a few, including fluoxetine and bupropion, inducing weight loss 68, 69 putatively through BDNF signalling. The mechanism of the effect of antidepressants on weight is still a matter of controversy. However, negative interaction occurs between impaired glucose tolerance and circulating BDNF levels such that serum BDNF levels are significantly decreased in Type 2 diabetes patients compared to normal controls 70. Moreover, selective BDNF infusion into the lateral ventricles of the brain decreased food intake and associated excessive weight gain in rat 71. Conversely, though other studies suggest that BDNF does not play an essential role in the regulation of energy expenditure 72, BDNF‐deficient mice show hyperphagia and consequent obesity 73. Caloric restriction therefore should reduce body weight and hyperglycaemia not only because of the reduced food intake *per se*, but also the biochemical consequences of the increase in BDNF level. BDNF enhances the sensitivity and hypoglycaemic action of insulin (Table 2) 74.

**Table 2:** Diet supplementation with antidepressant‐like effects

Supplement	Effect/mechanism of action	References
Fish oil	Reversed the depression‐altered, undesired lipid profiles and ghrelin level in serum	79
Aqueous extract of Channa (C.) striatus	Not fully elucidated	80
Ergothioneine	Promotes neuronal differentiation	22
Blueberry extract	Hypoglycaemic and anti‐peroxidative effects	81
Choline	Increased adult hippocampal neurogenesis and BDNF	82, 83, 84
Creatine	Not understood	85, 86
Omega‐3 fatty acids	Increase membrane fluidity and potentiates other antidepressants at subeffective doses	87, 88
n‐3 polyunsaturated fatty acids (PUFA), eicosapentaenoic acid (EPA) and docosahexaenoic acid (DHA)	Increased serum serotonin concentration, increased CREB phosphorylation and BDNF, and decreased hippocampal expressions of IL‐6 and TNFα.	23, 89, 90, 91
Taurine and beta‐alanine	Increased BDNF and hippocampal phosphorylation levels of ERK1/2, Akt, GSK3β, CREB and decreased metabolite of serotonin (5HTIAA)	[92, 93]
Perilla. frutescens seed oil‐rich	Increased BDNF and serotonin levels	19
Whole egg	Increased the Trp/LNAA ratio	2
Inulin‐type oligosaccharides	Not fully elucidated	[94]
Pre‐germinated brown rice	Decreased in frontal cortex 5‐HIAA/5‐HT ratio	[95]
Curcumin	Normalized levels of BDNF, synapsin I, and CREB and is an antioxidant.	[96, 97]
High‐fat diets (HFD)	Not fully elucidated	21

Many food substances or diet supplements have been found effective against animal models of depression. For example, dietary supplementation with fish products, including fish oil 75 and aqueous extract of Channa (C.) striatus (Malay‐Haruan) fillet, a freshwater snakehead fish consumed by local Malay population 76 produced significant reduction of immobility time in both FST and TST in mice. Fish oil supplementation not only provided antidepressant‐like effects but also reversed the altered lipid profiles and ghrelin level in serum 75. Ergothioneine is a hydrophilic antioxidant and contained at high levels in edible golden oyster mushrooms. It crosses the blood–brain barrier into the brain, when orally ingested, and promotes neuronal differentiation and alleviates symptoms of depression 22. Chronic consumption of blueberry extract exhibits antidepressant‐like and anti‐peroxidative effects in an animal model 77.

Some dietary supplements showed sex‐specific or age‐specific effects, providing insights into gender‐specific therapeutic target of antidepressant research. For example, supplemental dietary choline when given during development may inoculate an individual against depression 78 while 4% creatine supplementation displayed an antidepressant‐like response in female but not male rats 79, 80. Uridine or cytidine both of which stimulate synthesis of cytidine 5′‐diphosphocholine (CDP‐choline, a critical substrate for phospholipid synthesis) have antidepressant‐like effects in the forced swim test (FST) in rats. Moreover, dietary supplementation with omega‐3 fatty acids (OMG) which increase membrane fluidity and a subeffective dose of uridine produce synergistic antidepressant‐like effects 81, 82.

A long‐term dietary intake of *n*‐3 polyunsaturated fatty acids (PUFA) eicosapentaenoic acid (EPA) and docosahexaenoic acid (DHA) induces overexpression of CREB and hence produce antidepressant‐like effects in rats 83, 84. DHA is a key component of neuronal membranes at sites of signal transduction at the synapse, but mammals are inefficient in producing it, hence supplementation of DHA in the diet is important 23. In addition to reducing oxidative stress and inflammation, DHA serves to normalize levels of BDNF‐related synaptic markers in the hippocampus of brain injured animals 85. Similarly, high taurine‐supplemented diet for 4 weeks had an antidepressant‐like effect in the forced swim test in rodents 86, 87. Perilla frutescens seed oil‐rich diet exhibited antidepressant‐like properties through modulation of fatty acid profiles and BDNF expression in the brain 19. Nagasawa *et al*. suggest that whole egg may be an excellent food for preventing and alleviating the conditions of major depression, based on their experiments with rats: chronic whole‐egg administration increased the incorporation of tryptophan into the brain prefrontal cortex but no changes in serotonin level 2. Tryptophan/large neutral amino acid (Trp/LNAA) ratio is an index of the incorporation of tryptophan into the brain as LNAA compete with one another for the same amino acid transport system when they are incorporated into the brain. Dietary inulin‐type oligosaccharides extracted from Yacon (Smallanthus sonchifolius), a traditional food in the Andean diet, experimentally suggested to be a prospective natural source for antidepressants 88, while pre‐germinated brown rice increased in serotonin (5‐HT) levels in the mouse frontal cortex, producing antidepressant‐like effects in mice 89. Curcumin is a major chemical component of the turmeric plant (Curcuma longa), which has been widely used as a spice and food preservative in India for several generations. It has shown excellent efficacy in counteracting neuronal dysfunction in several models of neurodegenerative diseases in a process involving the action of the BDNF system 90, 91.

Despite the number of negative consequences of high‐fat diets (HFD) related to the impairment of hippocampal synaptic plasticity, changes in neurogenesis and decrease of glutamatergic transmission, De Rio *et al*. show that HFD triggers an antidepressant‐like action in the FST 21 independent of memory deficits. Going by other findings that HFD is a negative predictor of BDNF, this seems to be a controversial finding

## Concluding remarks

Caloric restriction, fasting or diet supplementation alone per se is unlikely going to be the ultimate solution to treating depression in humans. Despite the effectiveness in animal models, translation and application of these findings in humans with mental health problems are not straightforward. Keeping a healthy diet is in itself a challenge for people without mental health problems, let alone people with serious mental health problems. The most feasible solution, at least in the present and near future, is a combination of both dietary modification and antidepressant drugs. Moreover, most of the findings reviewed above admit that diet supplementation produced additive or synergistic effects when administered with conventional drugs or in combination with other interventions. For example, fasting produced additive effects with imipramine in mice 42 while lovastatin augmented the antidepressant‐like effects of fluoxetine in rats 92, 98 when given at a sub effective dose. Similarly, supplementation with omega‐3 fatty acids (OMG) and a subeffective dose of uridine produced synergistic antidepressant‐like effects, displaying a behavioural profile similar to fluoxetine 82 while 4% chronic creatine supplementation with fluoxetine produced a more robust antidepressant‐like behavioural profile compared to a dose of fluoxetine alone 80. The ability of energy restriction to up‐regulate BDNF expression and enhance neurogenesis in rodents suggests that it may also be possible to establish dietary regimens to enhance brain function and resistance to neurodegenerative diseases, including depression in humans, by controlling food intake and dietary manipulations 36, 53.

Most of the current antidepressant have adverse side effects at their effective doses, and any intervention that reduces their effective dosage is likely to reduce the side effects. Findings reviewed here raise the possibility that diet could be used to facilitate the effects of antidepressants in humans while reducing the side effects of current antidepressants. However, it is worth noting a caveat that some trials in animals have been done on normal, healthy animals. Whether the treatment would have positive effects in depressive phenotype remain to be seen. Nevertheless, caloric/dietary restriction, fasting and diet supplementation have a promising potential as adjuvant to antidepressants and lowering the adverse side effects, at least while a more lasting solution continues being sought. Therapeutic energy restriction has a lower cost and is easier to carry out than other treatments for drug‐resistant patients, such as electroconvulsive therapy 13.

## Conflict of interest

The authors declare no conflict of interests.
